# Does an Internet-Based Emotion Regulation Intervention Provide Added Value for Acute Psychiatric Inpatient Care? Protocol for a Randomized Controlled Pilot Trial

**DOI:** 10.2196/47656

**Published:** 2023-07-11

**Authors:** Laura Luisa Bielinski, Gwendolyn Wälchli, Christoph Nissen, Thomas Berger, Franz Moggi

**Affiliations:** 1 Department of Clinical Psychology and Psychotherapy University of Bern Bern Switzerland; 2 Division of Psychiatric Specialties Geneva University Hospitals Geneva Switzerland; 3 University Hospital of Psychiatry and Psychotherapy University of Bern Bern Switzerland

**Keywords:** blended treatment, inpatient, emotion regulation, internet intervention, acute psychiatric setting, randomized controlled pilot trial, randomized controlled trial, RCT, transdiagnostic, feasibility, emotion, mental health, psychiatric, psychiatry, randomized, internet based, digital health

## Abstract

**Background:**

There is a lack of studies on internet-based interventions in inpatient settings. This is especially true for studies of internet-based interventions in acute psychiatric inpatient care. Internet-based interventions in this specific setting may provide benefits such as patient empowerment and overall improved treatment outcomes. However, there may also be specific barriers to their implementation that are unique due to the complexity of acute psychiatric inpatient care.

**Objective:**

The aim of this study is to examine the feasibility and preliminary evidence for effectiveness of a web-based emotion regulation intervention provided as an add-on to acute psychiatric inpatient care.

**Methods:**

The goal is to randomly allocate 60 patients with a range of different diagnoses in a 1:1 ratio to either treatment as usual (TAU), which consists of acute psychiatric inpatient treatment, or to the intervention group, which will receive TAU plus access to a web-based intervention that focuses on reduction of emotion regulation difficulties and improvement of emotion regulation skills. The primary outcome is symptom severity, assessed with the short form of the Brief Symptom Inventory at baseline, after 4 weeks, after 8 weeks, and at hospital discharge. Secondary outcomes include 2 emotion regulation parameters, intervention use, usability, patient satisfaction, and reasons for patient loss to follow-up.

**Results:**

Participant recruitment started in August 2021 and as of March 2023 was ongoing. First publication of study results is expected in 2024.

**Conclusions:**

This study protocol describes a study that intends to examine a web-based emotion regulation intervention in acute psychiatric inpatient care. The study will provide information on the feasibility of the intervention and possible effects on symptom severity and emotion regulation. The results will provide new insights on blended treatment, in this case the combination of a web-based intervention and face-to-face psychiatric treatment, in an understudied patient group and setting.

**Trial Registration:**

ClinicalTrials.gov NCT04990674; https://clinicaltrials.gov/ct2/show/NCT04990674

**International Registered Report Identifier (IRRID):**

DERR1-10.2196/47656

## Introduction

Internet interventions are efficacious and effective for treating various mental health disorders [1-3]. In recent years, the combination of internet intervention and face-to-face treatment (blended treatment [4,5]) has been examined. Several studies point to the efficacy and effectiveness of blended treatment approaches in the outpatient setting for patients with depression [6-9]. Studies have also been conducted on outpatient blended treatment for anxiety disorders [10] and substance dependence [11,12]. Moreover, blended treatments with a more explicit transdiagnostic focus have also been examined in the outpatient setting. Examples include a treatment for emotional disorders [13] and transdiagnostic treatments with an emotion regulation focus [14]. However, only a few studies on blended treatment have been conducted with adult inpatients.

Several studies have focused on web-based and mobile-based interventions as aftercare after an inpatient stay [15-20]. For example, 2 studies evaluated aftercare interventions for eating disorders [16,17]. The first found a therapist-guided smartphone-based intervention showed high acceptance rates but nonsignificant effects on BMI and eating disorder symptoms [16]. The second found that a web-based program for women with bulimia nervosa applied as aftercare led to a significantly lower frequency of vomiting episodes but no significant differences in abstinence rates compared to treatment as usual (TAU) [17]. Another study investigated the effect of a self-help smartphone app on self-esteem among inpatients with depression after discharge from a psychiatric hospital [15]. While there was no significant improvement in the primary outcome (self-esteem) or secondary outcomes (depressive symptoms and quality of life), there was a small positive effect on self-competence in favor of the intervention group. Schlicker et al [18] examined a text message–based maintenance intervention for depression and determined whether individualized messages would lead to better outcomes than standardized messages that target emotion regulation. The results of the study suggested that standardized messages with an emotion regulation focus led to a significantly smaller increase in depressive symptoms than a waitlist condition. This was not the case for the condition with the individualized messages.

Regarding transdiagnostic interventions applied as aftercare in samples with mixed diagnoses, a study by Ebert et al [19] examined an internet-based maintenance intervention after inpatient treatment and found the intervention to be more effective than TAU with regard to the long-term outcomes of inpatient psychotherapy. Furthermore, a study by Zwerenz et al [20] was able to show that a group that received a psychodynamic web-based intervention provided after inpatient psychotherapy had improved depressive symptoms, quality of life, and emotional competence compared to a waitlist control group. However, completion rates for the program were low.

Regarding internet interventions applied during an inpatient stay, a few studies have focused on the feasibility of these types of interventions. Dorow and colleagues [21] examined an internet-based self-management program for depression, focusing on user acceptance and chances and barriers from both the patient and medical expert perspectives. User acceptance was reported to be moderate to high. Experts mentioned some aspects to be relevant barriers, such as technical problems, the severity of the course of depression, and the ability of patients to concentrate. Patients mentioned positive aspects of the intervention, such as ease of use and flexible availability, but also negative aspects, such as the design and structure of the program. Schwarz and colleagues [22] examined the feasibility of a tool for depressive disorders in inpatient care. They reported feasibility of the intervention but also low use rates. Moreover, general attitudes toward and acceptance of internet interventions by mental health professionals working in psychiatric hospitals have also been examined [23,24]. For example, a study by Sander and colleagues [23] showed that participants had little experience or knowledge about internet-delivered interventions. Their attitudes toward these types of interventions in inpatient care were indifferent. Participants mentioned benefits, such as optimized treatment structure and patient empowerment. They also mentioned anticipated barriers, such as patient symptom severity and insufficient technical equipment.

Even fewer studies have focused on the effectiveness of internet interventions during inpatient treatment. Zwerenz and colleagues [25,26] examined an internet intervention in an inpatient setting where patients with depression received the self-guided program as an addition to psychodynamic psychotherapy. The study showed a positive effect of the intervention (inpatient stay plus access to the internet intervention) on depressive symptoms with small to medium effect size at the end of the intervention (*d*=0.44) [25] and a medium effect size (*d*=0.58) at a follow-up assessment compared to an active control group [26]. Richter and colleagues [27] examined the same program that was investigated by Zwerenz and colleagues [25,26] but in a routine psychiatric inpatient setting with guidance added for the program. They found a medium-sized effect (*d*=–0.73) in favor of the intervention group, which received TAU plus access to the guided internet intervention, compared to the TAU group at the 12-week assessment timepoint and earlier discharge for patients who received access to the internet intervention compared to patients in the TAU condition. Finally, Bendig and colleagues [28] examined the feasibility of blended group training for social skills in inpatient acute psychiatric care. The blended treatment lasted 4 weeks and consisted of 4 face-to-face group sessions and 3 online modules. In the 1-group trial without a control group, the effect on social skills was not statistically significant, but the blended group training was deemed feasible for use in an acute inpatient setting.

Implementing an internet intervention in an acute psychiatric inpatient setting may offer benefits. On the one hand, the addition of an internet intervention to standard care or TAU may improve overall treatment effects, as has been demonstrated previously in outpatients and in studies focusing on inpatient treatment [6,25-27]. An internet intervention may thus provide a useful complement to treatment, one that is easily accessible during the inpatient stay, where patients may have idle timeslots during the treatment program. The few studies on an internet intervention in acute or routine psychiatric inpatient settings point toward the feasibility of such interventions [27,28].

However, implementing an internet intervention in acute psychiatric care may also come with specific barriers and complexities. Individuals in acute psychiatric inpatient care often enter treatment in acute crisis with severe disorders and with one or more comorbidities. A recent study by Becker and colleagues [29] showed that the number of diagnoses and symptom severity were associated with active or inactive participation in an online intervention during and after inpatient and day-clinic psychotherapeutic treatment. A lack of motivation due to symptom severity was also a reason for patients to decline participation in an inpatient study on e–mental health implementation by Van Assche et al [30]. According to Wentzel and colleagues [31], crisis risk and psychosocial problems are important factors to consider for a fit for blended treatment. Moreover, stays in acute wards are nowadays often short [28], and during this time, inpatients may already be saturated with other treatment components (eg, medication, psychotherapy, ergotherapy, and physiotherapy), making it difficult for patients to focus on an additional internet intervention. Wentzel and colleagues [31] also mention that having a private place to work is an important condition for blended treatment. This may not be available in an acute inpatient ward. Finally, all the different stakeholders involved in acute inpatient treatment may also make the implementation of such an intervention more complex [28].

The aim of this randomized controlled pilot trial is to investigate an internet-based intervention in acute psychiatric inpatient care. To this end, a web-based emotion regulation intervention (REMOTION; an acronym for “Regulate Emotion”) will be provided as an add-on to acute inpatient treatment in public psychiatric care at a university hospital. Different feasibility parameters will be recorded, including specific intervention parameters, such as use of the program and adherence, usability, satisfaction with the intervention, and reasons for patient loss to follow-up. Effects of the intervention will be assessed by comparing patients that receive access to REMOTION in addition to TAU to patients who receive TAU only on general symptom severity and emotion regulation difficulties and skills.

## Methods

### Study Design

**Figure 1:**
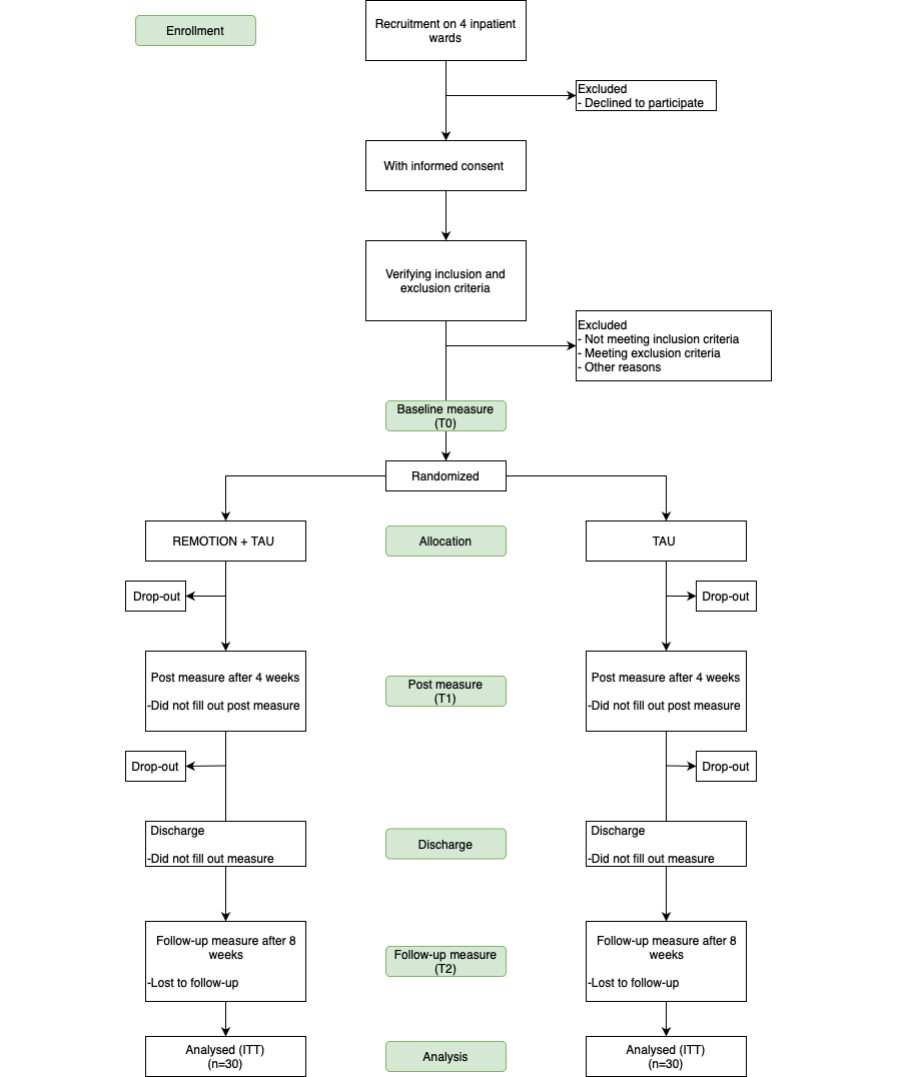
Planned study flow. Discharge can take place at any time after randomization. ITT: intention to treat; TAU: treatment as usual.

Sixty patients will be randomized in a 1:1 ratio to 2 trial arms: REMOTION plus TAU and TAU alone. Assessments will take place at baseline, after 4 weeks, after 8 weeks, and after discharge from inpatient stay (Figure 1). Assessments will take place irrespective of inpatient stay duration. The planned duration of the study is approximately 2 years, to reach 60 individuals. Since external validity and the generalizability of the results to acute inpatient care settings play an important role, the inclusion and exclusion criteria are broadly defined.

### Setting

The setting for the study is the acute inpatient psychiatric wards at the University Hospital of Psychiatry and Psychotherapy, Bern, Switzerland. The University Hospital of Psychiatry and Psychotherapy is part of the Universitäre Psychiatrische Dienste (UPD), Bern. The UPD guarantees psychiatric care in the Bern region, including adult and geriatric psychiatry, as well as child and adolescent psychiatry for the entire canton [32]. The University Hospital of Psychiatry and Psychotherapy treats patients with a variety of mental health diagnoses. For the study, patients are recruited from 4 different acute inpatient wards at the University Hospital of Psychiatry and Psychotherapy.

### Sample Size

Based on recommendations in the literature [33], the aim is to recruit a minimum of 30 participants per group during the trial. No power analysis is performed, as is commonly the case with randomized controlled pilot trials.

### Eligibility Criteria

To take part in the study, patients should fulfill the following criteria: (1) be older than 18 years, (2) be in acute inpatient treatment, (3) have access to the internet, and (4) provide written informed consent. The exclusion criteria include (1) participation in another specific intervention for emotion regulation; (2) a current episode or a history of psychotic disorders or bipolar disorder, intellectual disability, or organic mental disorders; (3) acute suicidality; and (4) not being fluent in German.

### Procedure

After consultation with the individual ward team at the weekly interdisciplinary ward meeting, individual patients are asked about study participation. Information on the study is provided and patients are asked if they would like to participate. Once written informed consent is provided, the inclusion and exclusion criteria are checked. Eligible patients are asked to complete the baseline questionnaires online. This takes approximately 15 to 20 minutes. Randomization occurs after baseline assessment. A computerized randomization procedure concealed from the study team is used to randomize patients. Patients randomized to the intervention group receive immediate access to REMOTION. Patients in TAU are informed that they can have access after the 8-week assessment, per request. There is no blinding implemented in the study. During the study period, the ward staff is informed about a patient’s designated trial arm. Patients receive a reminder to work on the program, either face-to-face from the study team if still on the ward or by telephone or email if discharge has already occurred, once a week for 4 weeks. For individuals who fail to complete follow-up assessments, interviews on reasons for loss to follow-up will be performed via telephone. Prior to study initiation, all 4 acute ward teams are informed in detail about the study, including the provision of a 1-hour workshop on the REMOTION intervention for each ward.

### Interventions

#### Control Group (TAU)

The active control group in this study receives TAU, comprising acute psychiatric inpatient treatment at the University Hospital of Psychiatry and Psychotherapy. This treatment consists of integrative, psychiatric-psychotherapeutic treatment. Depending on the individual conditions, it includes pharmacotherapy informed by current guidelines and adapted to individual needs, psychotherapy, contact with psychiatric nursing, specialist therapies such as occupational therapy or music therapy, and contact with social workers. The ward teams are informed not to talk about REMOTION content with patients in the control group. Individuals in the control group receive access to the intervention on request after the 8-week mark.

#### Intervention Group (REMOTION and TAU)

Patients in the intervention arm are given access to REMOTION in addition to TAU on the acute inpatient ward. The web-based intervention REMOTION was developed at the University of Bern by authors LLB and TB with input from author FM. It is structurally based on the extended process model (EPM) of emotion regulation [34]. It is currently also being examined as an add-on to outpatient psychotherapy in a different study [35] and is being examined in a large, randomized, controlled, 3-armed outpatient trial in Germany that focuses on effectiveness [36]. It has never been examined in an inpatient setting up to this point. The intervention contains an introduction module and 5 further modules: psychoeducation, identification, selection, implementation, and monitoring/flexibility [35]. The intervention’s aim is to reduce symptom severity and improve patient emotion regulation. As such, it does not target specific mental health disorders but is meant to be applied transdiagnostically, across disorders. Such an intervention may be of particular interest for inpatient populations, where comorbidity is likely highly prevalent [37]. A full overview of the intervention content for each module can be found in Table 1 and has also been published in 2 previous publications [35,36]. REMOTION is only available in German. The intervention runs on a server from the University of Bern and access is strictly password protected. Individuals in the intervention group immediately receive access to REMOTION and are asked to work on content for 4 weeks (1 to 2 modules a week). The study team reminds patients to work on the content once a week face-to-face if patients are still on the ward or by telephone or email if already discharged. Patients on the 4 wards have access to their smartphones, laptops, and tablets during their inpatient stay. Moreover, each ward is equipped with a ward computer that patients can use to engage with the program throughout their stay, if desired.

**Table 1:** REMOTION modules and content. Note that this entire textbox is adapted from tables already published in previous publications by Bielinski et al [35] and Trimpop et al [36], which are published under Creative Commons Attribution 4.0 International License [38].

Module	Content of module
Introduction	This module provides information on the content of the intervention and instructs patients on how to use the intervention.
Psychoeducation	This module provides psychoeducational content on emotions, their function, and types of emotional experience. It also introduces the concept of emotion regulation and its relationship to mental illness.
Identification	Emotional awareness (the perception substep of the identification stage of emotion regulation [34]) is explored in this module. The value of emotion regulation and information on if and when to regulate emotions is provided in this module.
Selection	In the selection module, patients learn about the types of emotion regulation strategies that are available. Situation selection or modification, attentional deployment, change of cognition, and response modulation are introduced to patients [39]. Furthermore, strategies specific to over- and underregulated states [40-42] are described.
Implementation	In the implementation module, patients learn how the strategies from the previous module can be implemented. Exercises are introduced for every emotion regulation strategy, and application of strategies in daily life is discussed.
Monitoring/flexibility	In the monitoring/flexibility module, patients are motivated to flexibly use emotion regulation strategies. Modifying strategies, maintaining, switching, and stopping [34,43] are discussed. Patients are encouraged to apply strategies to different contexts, to practice, and to try sequences or blends of strategies.

### Measures

#### Overview

All measures will be assessed via the internet, except for patient diagnoses at intake and medication at discharge, which will be retrieved from the hospital’s patient records. Interviews with patients will be conducted via telephone, and interviews with ward teams will be conducted face-to-face or via telephone. At baseline assessment, several demographic and other items will be assessed. These include questions on age, gender, nationality, mother tongue, highest degree of education, employment status, civil status, household size and composition, income, previous inpatient stays, previous experience with emotion regulation interventions, current involvement in an emotion regulation intervention, current involvement in other online therapy interventions, and frequency of use of online media. Study feasibility parameters, such as number of individuals approached for written informed consent, number of individuals with written informed consent, number randomized, and number of patients who completed all questionnaires, will be recorded for the study flow and presented in the main outcome paper to inform other institutions on the feasibility of the study design.

#### Primary Outcome Measure

The short version of the Brief Symptom Inventory (BSI-18) [44-46] is used to assess the primary outcome: general symptom severity. The BSI-18 contains the Global Severity Index (GSI), including all 18 items, as well as the three 6-item scales Somatization, Depression, and Anxiety [45]. The internal consistency is considered good for all 4 scales: Somatization (α=.82), Depression (α=.87), Anxiety (α=.84), and GSI (α=.93) [45]. The items are assessed on a 5-point Likert scale ranging from 0=“not at all” to 4=“very strong.” The BSI-18 is presented to patients at baseline assessment, after 4 weeks, at discharge, and after 8 weeks.

#### Secondary Outcome Measures

##### Emotion Regulation

Emotion regulation is assessed with 2 different questionnaires at baseline, after 4 weeks, after 8 weeks, and at discharge. One of the two questionnaires is the Emotion Regulation Skills Questionnaire (Fragebogen zur standardisierten Selbsteinschätzung emotionaler Kompetenzen; SEK-27) [47]. The questionnaire assesses emotional skills and includes a total of 27 items. The items are assessed on a 5-point Likert scale ranging from 0=“not at all” to 4=“almost always.” Studies have found good reliability and validity for the German and English versions of the questionnaire [48,49]. The Cronbach α is between .68 and .81 for the subscales and .90 for the total scale [47].

The second questionnaire is the Difficulties in Emotion Regulation Questionnaire (DERS) [50]; the study uses the German version by Ehring et al [51]. The DERS was developed to assess emotion regulation difficulties. It includes 6 subscales: lack of emotional awareness (Awareness), lack of emotional clarity (Clarity), nonacceptance of emotional responses (Nonacceptance), limited access to emotion regulation strategies (Strategies), difficulties engaging in goal-directed behaviors (Goals), and impulse control difficulties (Impulse) [50]. These subscales and the overall DERS score have high internal consistency (α=.93) [50].

##### System Usability

The System Usability Scale (SUS) is a 10-item scale giving a global view of subjective assessments of system usability [52]. The questionnaire asks users to rate their level of agreement with statements covering a range of usability characteristics, such as the complexity of the system and any support or training participants feel they need to use the system effectively. The evaluation of the questionnaire results in a usability score in the range of 0 to 100. Values greater than 68 typically indicate good usability [53]. The questionnaire will be provided to individuals in the intervention group after 4 weeks, after 8 weeks, and at discharge.

##### Patient Satisfaction

Patient satisfaction will be assessed with the Client Satisfaction Questionnaire (CSQ-8 [54]; the German version is the Fragebogen zur Patientenzufriedenheit; ZUF-8 [55]). Eight items are used to measure general satisfaction with aspects of the treatment received. The CSQ-8 is suitable for economic screening of patient satisfaction The CSQ-8 consists of 8 items that are formulated as questions; each has 4 predefined answer options without a neutral position (eg, excellent, good, less good, and poor) [55]. The CSQ-8 proves to be a reliable (Cronbach α=.90) 1-dimensional scale [56] and has validity regarding detailed quality assessment of the service and treatment outcome [56]. For this study, the CSQ-8 was adapted for an internet intervention (as has been done in previous studies [57,58]). The questionnaire will be provided to individuals in the intervention group after 4 weeks, after 8 weeks, and at discharge.

##### Program Use

Program use will be assessed by number of modules completed at 4 weeks and 8 weeks, number of exercises completed at 4 weeks and 8 weeks, and time spent in the program at 4 weeks and 8 weeks. These use parameters will be assessed for the intervention arm only.

##### Interviews With Patients Lost to Follow-Up

Telephone interviews are conducted with patients who are lost to follow-up. The purpose is to explore reasons why patients have not continued with the intervention and have not completed the questionnaires. The authors designed questions specific to the study. Interviews with patients lost to follow-up will be conducted by phone and audio recorded.

#### Other Measures

##### Interviews With Acute Inpatient Ward Staff

During the study, interviews with staff members from 3 of the 4 acute ward teams will be conducted. These interviews will occur at the earliest a few months after study commencement for teams to have sufficient experience with the intervention. Purposive sampling will be applied. A minimum of 1 member of the following stakeholder groups per ward will be interviewed: physicians, psychologists, and nurses, and if applicable, social workers and other stakeholder groups. Ward staff will provide written informed consent, fill out a short demographic data questionnaire, and be interviewed using a semistructured interview developed specifically for the study. Interview questions focus on expectations and attitudes toward blended treatment in acute inpatient care, specific experiences with the web-based intervention examined in this study, and the implementation of internet-based interventions in acute inpatient care. The analyses of these interviews will be published in a separate publication to the main outcome publication.

### Planned Analysis

Feasibility parameters (use, usability, and patient satisfaction) will be analyzed descriptively. Means, SD, and CIs will be reported for continuous data and counts or percentages will be reported for nominal data. The recorded interviews of patients lost to follow-up will be transcribed according to rules specified by Dresing and Pehl [59]. Patient interviews will then be analyzed with a content analysis approach [60]. The focus will be on the coding process to build categories where similarities and differences between patients will be explored [60]. MAXQDA (2022 version; VERBI Software) will be used for the analysis.

To examine the effects of the intervention, means and SDs for the primary and secondary outcomes will be reported at baseline (T0), at the end of the intervention (4 weeks; T1), at inpatient discharge, and at follow-up after 8 weeks (T2). Within- and between-groups effect sizes will be calculated using guidelines specified by Cohen [61].

Data will also be examined using inferential statistics. An intention-to-treat approach will be used. All patients who were initially assigned after randomization will be included. In a first step, baseline differences between the intervention group and the control group will be examined for all relevant variables. For metric variables, this is done using a 2-tailed *t* test for independent samples. For nominally scaled data, the chi-square test is used. In case of meaningful differences, those variables will be included in further analyses and controlled accordingly. In a second step, mixed models with “group” as the between-group factor and “time” as the within-group factor will be used to examine differences between groups. In a first analysis, a model including the study timepoints (eg, baseline, after 4 weeks, and after 8 weeks) will be calculated. In addition, an analysis including the discharge timepoint in the model will also be calculated. Specifically, the interaction between time and group will be of interest. The significance level is set at 5%.

Interviews with ward staff members will be audio-recorded and then transcribed using the guidelines specified by Dresing and Pehl [59]. Transcripts will be analyzed using thematic analysis [62] to examine ward staff experiences with the REMOTION intervention. MAXQDA software will be used for the analysis.

All quantitative analyses will be performed with the most current versions of R (R Core Team) and SPSS (IBM Corp).

### Ethical Considerations

The study has been approved by the Ethics Committee of the Canton of Bern, Switzerland (2020-01139). Informed consent is obtained from all participants. The trial is registered with clinicaltrials.gov (NCT04990674). The study will be conducted according to local regulations and the Declaration of Helsinki. The CONSORT-EHEALTH (Consolidated Standards of Reporting Trials for e-Health) [63] checklist will be followed in reporting the trial. Participants receive no compensation for taking part in the trial.

## Results

Recruitment for the study started in August 2021. As of March 2023, it was still ongoing. Results for the study are expected in 2024.

## Discussion

### Expected Findings

While a few trials on blended treatment in inpatient care exist, patients in the acute psychiatric inpatient setting are understudied, and there is a need for the examination and evaluation of internet-based interventions in this specific setting. This randomized controlled pilot trial explores a web-based emotion regulation intervention in this treatment setting. The study is designed to obtain information on the feasibility of the intervention. It is also designed to provide estimates of the effectiveness concerning reduction of symptom severity and improvement of emotion regulation. The study will inform future trials on blended treatment (ie, the combination of internet-based interventions with face-to-face treatment) in acute psychiatric inpatient care and thus provide evidence on the potential added value of a web-based intervention in this specific setting.

### Limitations

This is a randomized controlled pilot trial, and the targeted sample size is small. All estimations of possible effects on symptom severity and emotion regulation are thus preliminary only. The focus of the study lies with feasibility. As there are only a few studies on internet interventions in acute psychiatric care, the study is inherently exploratory. Several components of the specific acute psychiatric inpatient care setting may be challenging for study feasibility: recruitment of more severely impaired patients, fast turnaround on wards, the possibility of conversations between patients from different trial conditions on one ward, and multiple stakeholders being involved in treatment. We have tried to consider these factors by providing an adequate study duration; informing ward staff in detail about the study prior to commencement, including the fact that the TAU group should not receive information on REMOTION content; and by incorporating weekly reminders to work on the intervention, even if patients have been discharged.

### Conclusion

This randomized controlled pilot study is the first to examine a web-based emotion regulation intervention, REMOTION, in acute psychiatric inpatient care at a public university hospital. This type of blended treatment is grossly understudied. The results of the study will provide information on the potential added value of such an intervention for patients with severe mental health disorders in acute psychiatric care.

## Abbreviations

BSI: Brief Symptom Inventory
CSQ: Client Satisfaction Questionnaire
DERS: Difficulties in Emotion Regulation Questionnaire
EPM: extended process model
GSI: Global Severity Index
REMOTION: Regulate Emotion
SEK: Fragebogen zur standardisierten Selbsteinschätzung emotionaler Kompetenzen
SUS: System Usability Scale
TAU: treatment as usual
UPD: Universitäre Psychiatrische Dienste
ZUF: Fragebogen zur Patientenzufriedenheit

## Data Availability

The data sets generated and analyzed during the study described in this protocol will be available from the corresponding author on reasonable request.
